# The Time Required to Estimate the Case Fatality Ratio of Influenza Using Only the Tip of an Iceberg: Joint Estimation of the Virulence and the Transmission Potential

**DOI:** 10.1155/2012/978901

**Published:** 2012-05-10

**Authors:** Keisuke Ejima, Ryosuke Omori, Benjamin J. Cowling, Kazuyuki Aihara, Hiroshi Nishiura

**Affiliations:** ^1^Department of Mathematical Informatics, Graduate School of Information Science and Technology, The University of Tokyo, 7-3-1 Hongo, Bunkyo-ku, Tokyo 113-8656, Japan; ^2^Department of Biology, Faculty of Sciences, Kyushu University, 6-10-1 Hakozaki, Higashi-ku, Fukuoka 812-8581, Japan; ^3^School of Public Health, The University of Hong Kong, Level 6, Core F, Cyberport 3, 100 Cyberport Road, Pokfulam, Hong Kong; ^4^Institute of Industrial Science, The University of Tokyo, 4-6-1 Komaba, Meguro-ku, Tokyo 153-8505, Japan; ^5^PRESTO, Japan Science and Technology Agency, Saitama 332-0012, Japan

## Abstract

Estimating the case fatality ratio (CFR) of a novel strain of influenza virus during the early stage of the pandemic is one of key epidemiological tasks to be conducted as rapid research response. Past experience during the epidemics of severe acute respiratory syndrome (SARS) and influenza A (H1N1-2009) posed several technical challenges in estimating the CFR in real time. The present study aimed to develop a simple method to estimate the CFR based on readily available datasets, that is, confirmed cases and deaths, while addressing some of the known technical issues. To assess the reliability and validity of the proposed method, we examined the minimum length of time required for the assigned CFR to be included within the 95% confidence intervals and for the estimated CFR to be below a prespecified cut-off value by means of Monte Carlo simulations. Overall, the smaller the transmission potential was, the longer it took to compare the estimated CFR against the cut-off value. If policymaking and public health response have to be made based on the CFR estimate derived from the proposed method and readily available data, it should be noted that the successful estimation may take longer than a few months.

## 1. Introduction

When a new infectious disease emerges, the case fatality ratio (CFR) informs how lethal the infection or the disease is, measuring the virulence of the novel infection as the conditional probability of death given infection or disease [1, 2]. To understand the severity of infection, assess the impact of clinical and public health interventions, and anticipate the likely number of deaths in the population given the total number of infected individuals, quantifying the CFR in real time during the early stage of an epidemic is of utmost importance.

Among various uses of the CFR, the present study focuses on influenza, and in particular, the epidemiological determination of the severity in relation to epidemiological indices, such as the Pandemic Severity Index (PSI) in the United States [3]. As a process of public health policymaking, this index is used as a scientific criterion to choose and determine public health countermeasures, and thus, the CFR is regarded as key information for policymaking [4]. For instance, if the estimated CFR exceeds a prespecified reference value of 2.0%, which is sometimes quoted as the estimate of the CFR for the H1N1-1918 pandemic (Spanish influenza) [5], the PSI suggests that the government should recommend and implement all the nonpharmaceutical interventions listed, including voluntary isolation of clinically ill individuals at home, quarantine of household contacts, and social distancing [3].

However, while the CFR is theoretically calculated as a proportion of deaths to infected individuals, the actual calculation practice involves several technical problems owing to a few epidemiological features.

First, one cannot directly count all infected individuals during the course of an epidemic due to unobservable nature of infection, and commonly available datasets may be only confirmed cases through surveillance efforts. Moreover, the mild nature of influenza involves multiple steps of bias including ignorance of asymptomatic and subclinical infections, case ascertainment bias, imperfectness of a diagnostic testing method, and reporting bias. In fact, approximately 10% of confirmed infections with influenza (H1N1-2009) in households were shown to be fully asymptomatic [6, 7]. To partly address the issue of underascertainment, a technical advancement in synthesizing epidemiological evidences enabled us to estimate the symptomatic case fatality ratio (sCFR), the proportion of deaths to all symptomatic cases [8], although the denominator data are frequently based on nonspecific disease information such as influenza-like illness. As an alternative, a real-time serological study could potentially offer the denominator based on all infected individuals [9], but the seroepidemiological survey is costly and the diagnostic performance of serological testing in relation to the estimation of the CFR has yet to be fully clarified. While specific CFRs using confirmed cases and symptomatic cases as the denominator have been expressed as cCFR and sCFR, respectively, among studies of H1N1-2009 [2], the present study consistently uses the simplest notation “CFR,” intending it to represent the risk of death among all infected individuals (and so may be abbreviated as iCFR when necessary).

Second, the real-time estimation of the CFR has to take into account the time delay from illness onset to death and, thus, requires us to employ an appropriate statistical method to address censoring. This point must be considered, because all the cases are not fully exposed to the risk of death at a point in time during the course of an epidemic, and a simple ratio of the cumulative numbers of deaths to cases can yield biased (mostly, underestimated) CFR [10–13]. Third, it is critical to always keep it in mind that the risk of death is heterogeneous. In particular, the higher risk of influenza death than healthy adults is seen among those with underlying health conditions [14], including chronic obstructive pulmonary disease, asthma, pregnancy, chronic kidney failure, and diabetes. Perhaps reflecting this feature, the CFR clearly differs by age with the highest estimate among elderly and infants and the lowest among school-age children and young adults [15, 16]. Fourth, during an early stage of a pandemic, the number of deaths still remains very small and so the estimation of CFR suffers from broad uncertainty. Given that the CFR of influenza is likely to be small, and suffers from wide uncertainty, it is fruitful to clarify the minimum number of cases that are required to determine if the estimated CFR in real time is significantly below a prespecified cut-off value such as 2.0%.

While directly addressing clinically mild features and case ascertainment bias calls for synthesizing epidemiological evidence, for example, by employing hierarchical modeling approach [8], it is also important to clarify what can be done with readily available epidemiological information, such as confirmed cases and confirmed deaths. In the present study, we aim to propose a method to estimate the CFR based on the limited epidemiological data during the early stage of an epidemic. Through this investigation, we also aim to clarify the minimum number of days that are required to explicitly compare the estimated CFR to predetermined cut-off values of the CFR.

## 2. Materials and Methods

### 2.1. Assumptions

For clarity, here we describe the underlying epidemiological assumptions and settings. First, we focus on the early stage of a pandemic and ignore the depletion of susceptible individuals during this particular time period in which the number of newly infected individuals *i*(*t*) increases exponentially; that is, we focus on the log-linear phase alone for simplicity. Second, in realistic settings, *i*(*t*) cannot be directly counted as a function of time, and it is possible to observe only the confirmed cases, *c*(*t*). Third, during the early epidemic phase, a constant factor, *k* which scales the ratio of confirmed cases to all infected individuals, is assumed to remain a constant. In other words, we assume that the frequency of confirmed diagnosis among infected individuals does not vary over time. Fourth, we assume that the time delay from infection to death is independently and identically distributed and denotes the conditional probability density function as *f*(*s*) of length *s* days since infection. Moreover, among the confirmed cases, we assume that all death counts are recorded over time through surveillance system. Except for being reflected in the generation time distribution and the reproduction number, the event of death is assumed to be independent of the process of renewal. Finally, we consider a public health setting in which the time of emergence (or the time to initiate exponential growth) *t*_0_ is known (even approximately) as was the case in a specific epidemic study in which the starting time point of an epidemic was estimated [17, 18]. In the next subsection, we describe the estimation procedure of only a homogeneous population. However, the estimation problem of a population with heterogeneous risks of infection and death is discussed in Section 3.

### 2.2. Model Structure

We first describe the model structure deterministically. Throughout the paper, we ignore demographic stochasticity in infection process (see Section 4). Let *i*(*t*) be the incidence of infection at calendar time *t*. Also, let *t*_0_ be the time at which an epidemic starts with a single index case. Then *i*(*t*) increase exponentially as follows:

(1) $$i\left(t\right)=\exp\left\{r\left(t-t_{0}\right)\right\},$$

where *r* is the exponential growth rate of incidence. It should be noted that *r* may be referred to as the intrinsic growth rate, if the incidence data in question deals with only the very early exponential growth phase of an epidemic. However, as long as we consider the exponential growth as crude approximation of the epidemic curve, the exponential growth phase can be longer than that governed by the intrinsic growth rate and *r* is not restricted to be the very early epidemic phase (e.g., as was practiced previously [19, 20]). Let *p* be the CFR among all infected individuals. Assuming that the conditional probability density function of the time from infection to death *f*(*s*) is known, the number of deaths *d*(*t*) is modeled as

(2) $$d\left(t\right)=p\overset{\infty }{\underset{0}{\int }}i\left(t-s\right)f\left(s\right)ds,$$

which can be rewritten as

(3) $$\begin{array}{cc} d\left(t\right) & =p\exp\left\{r\left(t-t_{0}\right)\right\}\overset{\infty }{\underset{0}{\int }}\exp\left(-rs\right)f\left(s\right)ds\\ & =p\exp\left\{r\left(t-t_{0}\right)\right\}M\left(-r\right), \end{array}$$

where *M*(−*r*) represents the moment-generating function of the time from infection to death given the exponential growth rate *r*. One may integrate both sides and use the cumulative number of deaths by day *t*, *D*(*t*) for the estimation of CFR. The estimator of the CFR is then given by

(4) $$\hat{p}=\frac{D\left(t\right)}{\left[\exp\left(r\left\{t-t_{0}\right\}\right)-\exp\left(-rt_{0}\right)\right]\left(M\left(-r\right)/r\right)}.$$

Other than parameters for *f*(*s*), which we will assume as known, the estimator (4) indicates that, to estimate the CFR, an unknown parameter *r* has to be estimated from an additional series of data other than the death process, for example, from the confirmed case series. The exponential growth rate *r* quantifies the denominator of the above-mentioned estimator. To estimate *r*, we analyze the incidence of confirmed cases, *c*(*t*). Let *l* denote the proportion of confirmed cases to the total of infected individuals the data-generating process of *c*(*t*) is described by

(5) $$\begin{array}{cc} c\left(t\right) & =l\overset{\infty }{\underset{0}{\int }}i\left(t-s\right)h\left(s\right)ds\\ & =lQ\left(-r\right)i\left(t\right)\\ & =ki\left(t\right), \end{array}$$

where *h*(*s*) is the density function of the time from infection to confirmatory diagnosis and *Q*(−*r*) represents the moment-generating function given the exponential growth rate *r*. We refer to the parameter *k* as the confirmed coefficient (i.e., *k* = *lQ*(−*r*)) which acts as a constant factor to translate *i*(*t*) into *c*(*t*).

Let us consider an adjusted calendar time based on known *t*_0_ (i.e., *t* + *t*_0_), we simplify all the following equations by eliminating *t*_0_ (and hereafter we consistently use *t* as the adjusted time in which *t*_0_ is equated to be zero). In addition to this adjustment, we discretize both the series of confirmed cases and deaths, because the observed dataset is given with discrete time (i.e., daily data), that is,

(6) $$\begin{array}{cc} c_{t} & =\overset{t}{\underset{t-1}{\int }}c\left(x\right)dx\\ & =\frac{k}{r}\left[\exp\left(rt\right)-\exp\left\{r\left(t-1\right)\right\}\right],\\ d_{t} & =\overset{t}{\underset{t-1}{\int }}d\left(x\right)dx\\ & =p\left(\exp\left(rt\right)-\exp\left\{r\left(t-1\right)\right\}\right)\frac{M\left(-r\right)}{r}. \end{array}$$

The proposed estimation method based on these linear approximations would work even when the exponential growth rate varies with time (e.g., varies as a step function) as was considered when evaluating the effectiveness of public health interventions such as school closure [21, 22]. The number of parameters would have to increase to capture the time variation (e.g., from a single *r* alone to *r*_0_ and *r*_1_ for two consecutive epidemic phases), and thus the required sample size for estimation would also increase. However, all we have to do to cope with the time dependence is to update (1) and (2) using multiple growth rates and, thus, revise (6), accordingly. 

### 2.3. Maximum Likelihood Estimation

Assuming that the observed number of confirmed cases on day *t* results from Poisson sampling process with mean *c*_*t*_ = *k*  [exp⁡(*rt*) − exp⁡{*r*(*t* − 1)}]/*r*, where *r* and *k* are parameters, the likelihood function is given as follows:

(7) $$L_{1}\left(r,k;m_{t}\right)=\overset{T}{\underset{t=1}{\prod }}\frac{{\left(k/r\right)}^{m_{t}}{\left[\exp\left(rt\right)-\exp\left\{r\left(t-1\right)\right\}\right]}^{m_{t}}\exp\left[-\left(k/r\right)\left[\exp\left(rt\right)-\exp\left\{r\left(t-1\right)\right\}\right]\right]}{m_{t}!},$$

where *m*_*t*_ is the observed daily number of confirmed cases on day *t* and *T* represents the latest time of observation.

Let *π*_*t*_ be a random variable which yields an estimator of the CFR on day *t* since the start of an epidemic and is the realized value in the particular epidemic. Assuming that the realized CFR is the result of binomial sampling process of death with sample size [exp⁡(*rT*) − 1]*M*(−*r*)/*r*, the likelihood to estimate the CFR based on the total number of deaths up to the latest time of observation *T* is

(8) $$\begin{array}{cc} L_{2}\left(\pi_{T};D\left(T\right),r\right) & =\left(\begin{array}{c} \left(\exp\left(rT\right)-1\right)\frac{M\left(-r\right)}{r}\\ D\left(T\right) \end{array}\right){\pi_{T}}^{D(T)}\\ & \;\times {\left(1-\pi_{T}\right)}^{\left(\exp(rT)-1)(M(-r)/r)-D(T\right)}. \end{array}$$

Because of an assumption of conditional independence between the renewal process and death, the total likelihood *L* is given by

(9) $$L=L_{1}L_{2}.$$

Minimizing the negative logarithm of the total likelihood *L*, we jointly estimate three parameters, *π*_*T*_, *r*, and *k*. The 95% confidence intervals (CIs) are derived from the profile likelihood, the idea of which is to invert a likelihood ratio test to obtain a CI for the parameter in question [23]. 

### 2.4. Simulations

Whereas the above-mentioned estimation procedure enables us to estimate the CFR based only on the confirmed cases and deaths, the estimation rests on limited epidemiological information as compared to other methods involving additional symptomatic case data or serological dataset. Thus, it is important to examine if we can overcome uncertainty and realistically employ the proposed method during an early phase of a pandemic. Specifically, we explore the time required to confidently suggest the range of the CFR and compare the CFR against a pre-specified cut-off value during the early stage. We assess the reliability and validity by means of random simulations.

As a plausible parameter range, we examine three different exponential growth rates, *r*, of 0.05, 0.15, and 0.25 per day. These are chosen as plausible, because, assuming that the mean generation time of influenza is 3 days and exponentially distributed, the basic reproduction number ranges from 1.15 to 1.75. If the generation time is a constant 3 days, the reproduction number ranges from 1.16 to 2.11. These are in line with published estimates of the reproduction number for H1N1-2009 [24]. In fact, the growth rate of influenza A (H1N1-2009) is estimated as 0.08 [25] and 0.10 per day [18] in Japan and Mexico, respectively. The reference values of CFR, *p*, are set at 0.1%, 0.5%, and 2.0% that are in line with the PSI in the United States. The CFR of Spanish influenza is sometimes thought to be approximately 2.0% [5, 24] and those of Asian and Hong Kong influenza pandemics are thought to be up to 0.5% [26]. The CFR of seasonal influenza is thought to be below 0.1% [27]. Although the CFR of the H1N1-2009 pandemic among all infected individuals is estimated to be smaller than 0.1% [28], we do not examine smaller estimates of the CFR, because 0.1% may be most reasonably defined as the lowest cut-off value in practical setting to distinguish a mild influenza strain from severe ones, and it is likely to be infeasible to robustly estimate a CFR below 0.1% during the early epidemic phase due to sampling errors. Since the empirically estimated proportion of confirmed cases among all infected individuals is 5% [15], we fix *k* at 0.05 assuming that the time delay from infection to confirmed diagnosis is sufficiently short. Ignoring small delay from infection to illness onset (as it does not influence the above-mentioned estimation framework), the conditional probability density function of the time from infection to death of influenza A (H1N1-2009) *f*(*s*) is assumed to be gamma distribution with the mean and standard deviation being 9.5 and 4.7 days, respectively [29].

As for simulation-based assessment, we first perform Monte Carlo simulations for 1000 times for each specified combination of parameter values, calculating the coverage probability of including the assigned CFR value within the 95% confidence intervals. Second, we assess the time at which the estimated CFR is confidently said as smaller than the pre-specified CFR value. Again, the model is randomly simulated for 1000 times per each parameters setting, calculating the number of simulation runs in which the upper 95% confidence interval is below the reference CFR value.

### 2.5. Heterogeneous Population

The proposed method can be extended to a heterogeneous population with differential risks of infection and death, perhaps by age and risk groups. We present the extended model analytically and demonstrate that the above-mentioned approach is directly applicable to a multihost population and, thus, the age-stratified epidemiological data.

## 3. Results

### 3.1. Reliability

**Figure 1:**
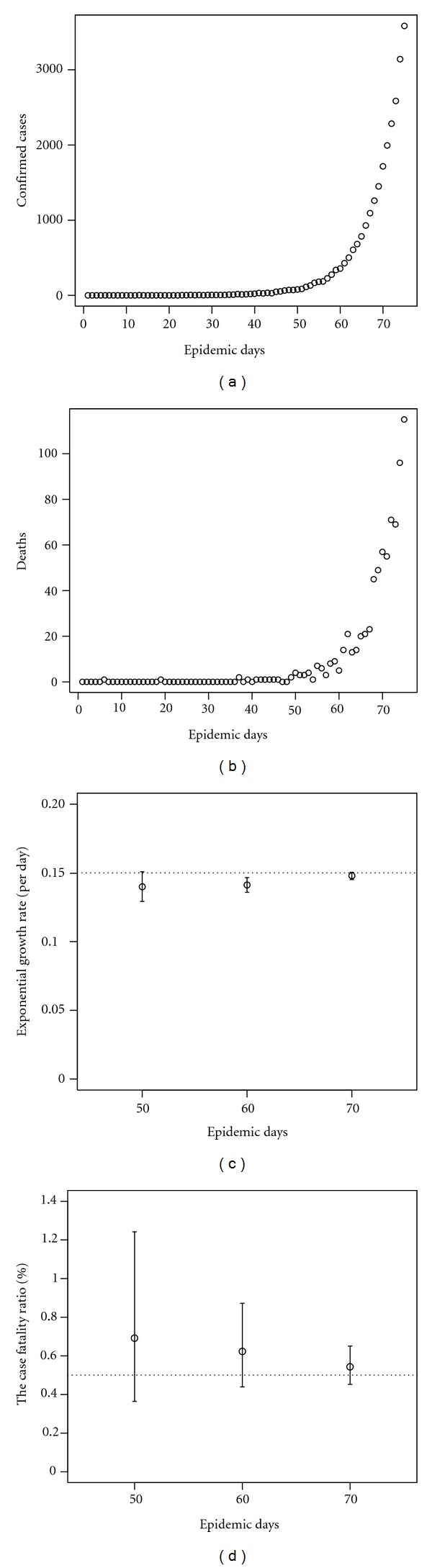
A single simulation run and the joint estimation results of the case fatality ratio and the exponential growth rate. The assigned CFR value is 0.5%, and the exponential growth rate *r* is set at 0.15 per day. ((a) and (b)) Incidence of confirmed cases and deaths as a function of epidemic days. The epidemic day 0 is the date on which an index case is infected. The numbers of confirmed cases and deaths increase exponentially. ((c) and (d)) The maximum likelihood estimates of (c) the exponential growth rate *r* and (d) the case fatality ratio with the 95% confidence intervals. The 95% confidence intervals were computed by employing the profile likelihood. Unfilled circles are the maximum likelihood estimates accompanied by the whiskers extending to lower and upper 95% confidence intervals. The dotted horizontal line shows the assigned parameter value.

Figure 1 illustrates a single simulation run and the resulting maximum likelihood estimates with the 95% confidence intervals with the assigned parameters, the CFR of 0.5%, and *r* = 0.15 per day. As one can imagine, the confidence intervals for each parameter are gradually narrowed down as the epidemic progresses due to reduced sampling errors. During the very early stage of the pandemic (e.g., for the first 40 days given the assumed parameters), it is not feasible to expect the narrow confidence interval for the CFR, and thus, one may fail to assess the reliability using only the very limited early epidemiological data.

Table 1 shows the coverage probability of the CFR for each set of parameters. When estimating the CFR based on early epidemic data, the exponential growth rate appears to play a critical role in determining reliability. To attain the coverage probability greater than 90% with *r* = 0.05, 0.15, and 0.25 per day, respectively, the latest times of observation, *T*, should be at least 80, 40, and 30 days with the reference CFR value of 0.5%. This indicates that the smaller the transmission potential is, the longer the time it would take to obtain a reproducible estimate of the CFR. Of course, the coverage probability converges to 95% with longer observation times. Given a larger CFR, the coverage probability converges earlier due to smaller sampling errors. However, the coverage probability appears to be more sensitive to variation in *r* than that in the CFR value.

### 3.2. Validity

The validity of comparing the CFR against pre-specified cut-off values is summarized in Table 2. The overall qualitative patterns are similar to those of the coverage probability in Table 1. The minimum number of days that is required to declare that the CFR is below cut-off values is very sensitive to the exponential growth rate of cases. In other words, smaller transmission potential requires us to wait for longer time to compare the estimated CFR against the cut-off values. In addition, the validity is also sensitive to the estimated CFR relative to the cut-off value. When the relative ratio of the CFR to the cut-off value gets smaller, the difficulty in differentiating the CFR is magnified. Given identical transmission potential and an identical assigned value of CFR, there was approximately a 20-day lag in the minimum numbers of days for differentiation between the relative case fatality ratios of 50% and 80%. With the smallest growth rate of *r* = 0.05 per day, the estimation framework failed to yield any successful differentiation of CFR, even observing the epidemic for *T* = 100 days. Of course, the estimated CFR also influences the feasibility, but the successful differentiation appears to be most sensitive to the exponential growth rate.

### 3.3. Heterogeneous Population

The proposed method is not directly applicable to realistic setting in which we observe substantial heterogeneities in the risks of infection and death. Accordingly, here we show the modeling approach to heterogeneous populations analytically. Specifically, we consider age-dependent dynamics: while the risk of infection may be higher among children than among elderly in the case of influenza, the conditional risk of death given infection is likely to be higher among elderly than school-age children, perhaps reflecting higher proportion of elderly with the underlying comorbidities.

Let *i*_*s*_(*t*) be the incidence of infection among subgroup *s* at calendar time *t*. Also, let *R*_*qs*_ be the average number of secondary cases in subgroup *q* generated by a single primary case in subgroup *s*, which would act as a single entry of the age-dependent next-generation matrix [30]. Assuming that the density function of the generation time *g*(*τ*) of length **τ** days is shared among subgroups, the multivariate renewal process is described by

(10) $$i_{s}\left(t\right)=\underset{q}{\sum }R_{sq}\overset{\infty }{\underset{0}{\int }}i_{q}\left(t-s\right)g\left(s\right)ds.$$

Let *p*_*s*_ be the group-specific CFR (e.g., age-specific CFR) among all infected individuals of subgroup *s*. As was shown with application to the homogeneous population, we employ the confirmed coefficient *k*_*s*_, reflecting both the proportion of confirmed cases to all infected individuals of subgroup *s* and the time delay from infection to confirmatory diagnosis. Then the confirmed cases among subgroup *s* *c*_*s*_(*t*) are

(11) $$c_{s}\left(t\right)=k_{s}i_{s}\left(t\right).$$

Since the observed dataset is discrete time series, that is, daily data, we integrate the confirmed cases as follows:

(12) $$c_{s,t}=\overset{t}{\underset{t-1}{\int }}c_{s}\left(x\right)dx.$$

Assuming that the conditional probability density function of the time from infection to death, *f*(*s*) is known and is shared among subgroups, the number of new deaths of subgroup *s* at time *t* *d*_*s*_(*t*) is described as

(13) $$d_{s}\left(t\right)=p_{s}\overset{\infty }{\underset{0}{\int }}i_{s}\left(t-s\right)f\left(s\right)ds,$$

which is rewritten as

(14) $$d_{s}\left(t\right)=p_{s}\overset{\infty }{\underset{0}{\int }}\underset{q}{\sum }R_{sq}\overset{\infty }{\underset{0}{\int }}i_{q}\left(t-\tau -s\right)g\left(\tau \right)d\tau f\left(s\right)ds.$$

As was integrated in the homogeneous case, one may focus on the cumulative number of deaths *D*_*s*_(*T*) by the latest time of observation *T*. The estimator of the group-specific CFR is given by

(15) $${\hat{p}}_{s}=\frac{D_{s}\left(T\right)}{\int_{0}^{T}\int_{0}^{\infty }\sum_{q}R_{sq}\int_{0}^{\infty }i_{q}\left(t-\tau -s\right)g\left(\tau \right)d\tau f\left(s\right)dsdt}.$$

The likelihood function to estimate the next-generation matrix may partially account for stochastic dependence structure of the transmission dynamics, and thus, conditions for every future expectation on the past history of the epidemic. Let **Z**(*t*) represent the history of age-specific confirmed cases from time 0 up to time *t* − 1. Given the series up to *t* − 1, and assuming that the incidence of confirmed cases on day *t* is sufficiently characterized by Poisson distribution, the conditional likelihood is written as

(16) $$\begin{array}{c} L_{1}\left(R_{ij},k;Z\left(t\right)\right) \end{array}=\underset{s}{\prod }\overset{n}{\underset{i=1}{\prod }}\frac{\exp\left(-\sum_{q}R_{sq}\int_{0}^{\infty }c_{q}\left(t-s\right)g\left(s\right)ds\right){\left(\sum_{q}R_{sq}\int_{0}^{\infty }c_{q}\left(t-s\right)g\left(s\right)ds\right)}^{m_{t,s}}}{m_{t,s}!},$$

where *m*_*t*,*s*_ represents the observed number of confirmed cases in subgroup *s* on day *t*. This likelihood function is useful to describe the underlying epidemic dynamics of the heterogeneous population. Let *π*_*t*,*s*_ be a random variable which yields an estimator of the CFR of subgroup *s* at day *t* since the start of an epidemic. The other likelihood to estimate *π*_*t*,*s*_ is assumed to be given by binomial sampling process as follows:

(17) $$\begin{array}{cc} & L_{2,s}\left(\pi_{s,T};D_{s}\left(T\right),R_{ij},k\right)\\ & =\left(\begin{array}{c} \overset{T}{\underset{0}{\int }}\overset{\infty }{\underset{0}{\int }}\underset{q}{\sum }R_{sq}\overset{\infty }{\underset{0}{\int }}i_{q}\left(t-\tau -s\right)g\left(\tau \right)d\tau f\left(s\right)dsdt\\ D_{s}\left(T\right) \end{array}\right)\\ & \;\times {\pi_{s,T}}^{D_{s}(T)}{\left(1-\pi_{s,T}\right)}^{\int_{0}^{T}\int_{0}^{\infty }\sum_{q}R_{sq}\int_{0}^{\infty }i_{q}(t-\tau -s)g(\tau )d\tau f(s)dsdt-D_{s}(T)}. \end{array}$$

Therefore, the total likelihood is calculated as the following product:

(18) $$L=L_{1}\underset{s}{\prod }L_{2,s}.$$

Although the estimation framework can thus be very similar to that for the homogeneous population, it should be noted that the validity and reliability of estimation procedure for the heterogeneous population are likely to be influenced by way of parameterizing the next-generation matrix. For example, if the quantification of the matrix requires us to estimate only a small number of parameters (e.g., one parameter for each age group), we expect that the validity and reliability are not too much different from those we examined for the homogeneous population. However, when more parameters should be estimated to describe more detailed underlying heterogeneous transmission dynamics, the proposed method has to face greater uncertainty.

## 4. Discussion

We proposed an estimation method to jointly infer the CFR and the exponential growth rate using only the confirmed case and death data. By means of Monte Carlo simulations, we assessed the minimum length of days required to compare the estimated CFR with the pre-specified CFR value such as those in the US Pandemic Severity Index. To do so, it appeared that the validity and reliability were very sensitive to the exponential growth rate and, thus, to the transmission potential of a novel pandemic strain. To be confident that the method included the CFR estimate within the 95% confidence interval, it appears that we have to wait at least for a month, and perhaps in general for about a few months given that the growth rate is equal to or smaller than 0.15 per day. The successful differentiation of CFR from cut-off values also takes about a few months. As it takes longer time for the estimation or differentiation, it would become more difficult for the incidence curve to be approximated by exponential growth. More importantly, the differentiation may not be feasible, if the growth rate is 0.05 per day or smaller. The growth rate was thus shown to play the most critical role in determining the feasibility of the proposed method than the CFR value to be estimated. This finding is attributable to the fact that the number of deaths is the result of binominal sampling of cases. In general, as the sample size (or the number of binomial trials) increased, the standard error of binomial probability decreased, and the number of binomial trials in the proposed model substantially increased as the exponential growth rate increased. The validity and reliability were more sensitive to the growth rate than the binomial probability: the influence of variation in binomial probability on its confidence interval was small for the assumed range of CFR values, which can be understood from the approximate standard error of the binomial probability derived from the normal approximation to binomial [31].

Already, there have been multiple epidemiological methods to estimate the CFR using different datasets. Presanis et al. [8] proposed a Bayesian evidence synthesis approach using various different types of data that describe a pyramid structure, explaining that confirmed cases represent the tip of an iceberg of infected individuals and emphasizing a need to observe milder fraction of cases such as those who attended medical service. The useful datasets for that method included medically attended symptomatic cases and those required hospitalization and those admitted to intensive care unit. Comparing our proposed method with the evidence synthesis approach, the proposed method has two important advantages: (i) the proposed method can comply with a need to offer the CFR estimate in real time and (ii) we use the time series data of the confirmed cases and deaths which are readily available and accessible. The two different methods may thus be combined and used in practical setting: while the proposed method is used for the real-time assessment using widely available data, the sCFR estimate employing the evidence synthesis approach may be subsequently offered based on datasets of well-defined cohort populations. On the other hand, Wu et al. [15] used real-time seroprevalence data during the course of an epidemic. This approach enables us to estimate the background denominator of incidence of infections directly. In fact, a seroepidemiological study may be the only method to explicitly and directly quantify the underlying transmission dynamics. However, seroepidemiological surveys are costly and explicit interpretations of seroconversion and changes in antibody titers have yet to be offered. As a method to supplement the explicit estimation approaches, we believe that the proposed method based on readily available data would be a useful real-time assessment tool.

There are a few important future tasks for improvement. First, we used no prior information of parameters in the present study, but some information may be retrieved from other datasets or from the literature including historical epidemic records (e.g., the exponential growth rate of the epidemic caused by the same infectious agent before conducting the estimation). In fact, it is frequently the case that the transmission potential or the growth rate of cases is estimated earlier than the CFR in practical setting, and one may know a plausible value of *r* in advance of CFR estimation. If the prior knowledge could compensate unknown information of the proposed method, it will help to reduce the associated uncertainty of the CFR, thereby improving the validity of estimation. Second, we did not take into account demographic stochasticity in the present study, but the stochasticity may not be negligible during the early epidemic phase [32–34]. The uncertainty that we quantified in the present study is likely to have been underestimated, although the qualitative findings are expected not to be different from those when we explicitly account for stochasticity using appropriate models (e.g., [32, 35]), especially for highly transmissible virus. Third, the proposed method as well as two earlier estimation studies based on evidence synthesis and serological study relied on the observed number of deaths as the numerator of the CFR. If there are many undiagnosed deaths, the direct estimation of the CFR is not feasible, and so, the virulence may also have to be assessed by indirect measurement such as that using excess mortality [36, 37]. Of course, constant *k* over time is also an unsupported assumption for epidemics with time-varying ascertainment efforts.

For a heterogeneous population we have shown that the proposed estimation framework for the homogeneous population can be easily extended to the heterogeneous setting. However, we have also discussed that the limited degrees of freedom might increase the relevant uncertainty; that is, when we consider *n* different subgroups, we have to deal with the next-generation matrix with *n* ^2^ entries in addition to *n* unknown parameters for the group-specific CFR, *p*_*s*_. Thus, the minimum length of days *T* that is required to estimate the CFR would be extended, and *T* would depend on the way we parameterize the next-generation matrix. Thus, we failed to offer simulation results with general conclusions with respect to the validity and reliability for the heterogeneous population. Given that the transmission of H1N1-2009 has been highly dependent on age [18, 32, 35, 38], one will have to balance the detailed descriptions of dynamics involving many subpopulations with the uncertainty surrounding the joint estimation of the CFR and the transmission potential.

If policymaking and public health response have to be made based on the real-time estimate of the CFR, the proposed method can be employed using only the readily available epidemiological datasets. However, as long as the estimation of the CFR relies on the proposed method, it should be noted that it may take longer than a few months to derive the CFR with sound uncertainty bounds, and thus, the very early response may not be able to base the policy decision on the CFR. Moreover, when the transmission potential is small, the number of infected individuals (or cases) may better be estimated directly from serological data (or medical attendance), because the proposed method is prone to uncertainties arising from low frequency of infection. While such limitation exists, we believe that the proposed method can be coupled with or supplement existing estimation frameworks which have to use additional epidemiological and serological data, especially for diseases with high transmission potential.

## Figures and Tables

**Table 1:** Coverage probability of the case fatality ratio (CFR) for each set of parameter values.

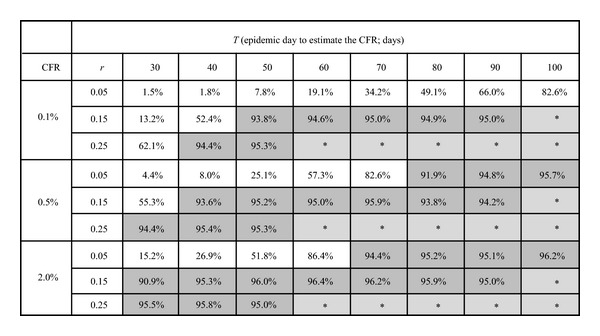

CFR: case fatality ratio (assigned value). All the values were calculated as the proportion of successful simulation runs with the 95% confidence intervals that include the assigned CFR value among the total of 1000 simulation runs. The parameter *r* is the exponential growth rate of infection (per day). *T* is the epidemic date on which the estimation is performed. Those exceeding the coverage probability of 90% are highlighted in dark grey, while the cells with *mark and shaded in light grey represent combinations of parameter values which generate too large numbers of cases and for which we refrained from estimation.

**Table 2:** Proportion of simulation runs in which the upper 95% confidential interval of CFR (*p*) falls below specified cut-off values.

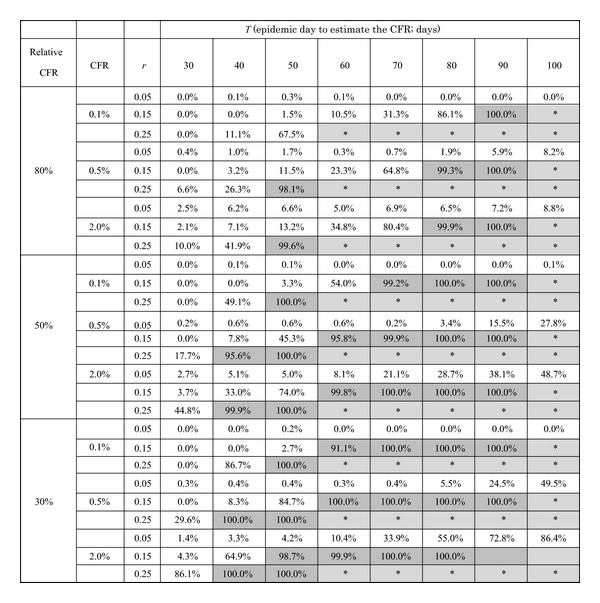

CFR: case fatality ratio (assigned value). Relative CFR: the ratio of assigned CFR value relative to the cut-off value. The proportion of successful simulation runs with the upper 95% confidence interval below the pre-specified cut-off value is shown. The parameter *r* is the exponential growth rate of infection (per day). *T* is the epidemic date on which the comparison is performed. Those exceeding the proportion of 90% are highlighted in dark grey, while the cells with *mark and shaded in light grey represent combinations of parameter values which generate too large numbers of cases and for which we refrained from estimation.
